# A novel computer-aided diagnostic system for accurate detection and grading of liver tumors

**DOI:** 10.1038/s41598-021-91634-0

**Published:** 2021-06-23

**Authors:** Ahmed Alksas, Mohamed Shehata, Gehad A. Saleh, Ahmed Shaffie, Ahmed Soliman, Mohammed Ghazal, Adel Khelifi, Hadil Abu Khalifeh, Ahmed Abdel Razek, Guruprasad A. Giridharan, Ayman El-Baz

**Affiliations:** 1 BioImaging Lab, Department of Bioengineering, University of Louisville, Louisville, KY 40292 USA; 2 Department of Radiology, Faculty of Medicine, Mansoura University, Mansoura, 35516 Egypt; 3 College of Engineering, Abu Dhabi University, Abu Dhabi, UAE; 4 Computer Science and Information Technology, Abu Dhabi University, Abu Dhabi, UAE

**Keywords:** Cancer imaging, Magnetic resonance imaging

## Abstract

Liver cancer is a major cause of morbidity and mortality in the world. The primary goals of this manuscript are the identification of novel imaging markers (morphological, functional, and anatomical/textural), and development of a computer-aided diagnostic (CAD) system to accurately detect and grade liver tumors non-invasively. A total of 95 patients with liver tumors (M = 65, F = 30, age range = 34–82 years) were enrolled in the study after consents were obtained. 38 patients had benign tumors (LR1 = 19 and LR2 = 19), 19 patients had intermediate tumors (LR3), and 38 patients had hepatocellular carcinoma (HCC) malignant tumors (LR4 = 19 and LR5 = 19). A multi-phase contrast-enhanced magnetic resonance imaging (CE-MRI) was collected to extract the imaging markers. A comprehensive CAD system was developed, which includes the following main steps: *i)* estimation of morphological markers using a new parametric spherical harmonic model, *ii)* estimation of textural markers using a novel rotation invariant gray-level co-occurrence matrix (GLCM) and gray-level run-length matrix (GLRLM) models, and *iii)* calculation of the functional markers by estimating the wash-in/wash-out slopes, which enable quantification of the enhancement characteristics across different CE-MR phases. These markers were subsequently processed using a two-stages random forest-based classifier to classify the liver tumor as benign, intermediate, or malignant and determine the corresponding grade (LR1, LR2, LR3, LR4, or LR5). The overall CAD system using all the identified imaging markers achieved a sensitivity of 91.8%±0.9%, specificity of 91.2%±1.9%, and F$_{1}$ score of 0.91±0.01, using the leave-one-subject-out (LOSO) cross-validation approach. Importantly, the CAD system achieved overall accuracies of $88\%\pm 5\%$, 85%±2%, 78%±3%, 83%±4%, and 79%±3% in grading liver tumors into LR1, LR2, LR3, LR4, and LR5, respectively. In addition to LOSO, the developed CAD system was tested using randomly stratified 10-fold and 5-fold cross-validation approaches. Alternative classification algorithms, including support vector machine, naive Bayes classifier, k-nearest neighbors, and linear discriminant analysis all produced inferior results compared to the proposed two stage random forest classification model. These experiments demonstrate the feasibility of the proposed CAD system as a novel tool to objectively assess liver tumors based on the new comprehensive imaging markers. The identified imaging markers and CAD system can be used as a non-invasive diagnostic tool for early and accurate detection and grading of liver cancer.

## Introduction

Liver cancer is the sixth leading cancer in the world with 800,000 new cases every year. It is the third most common cause of death worldwide with 700,000 deaths annually. Hepatocellular carcinoma (HCC) is the most common primary liver cancer accounting for more than 90% of cases^1^. On average, globally, one in every 5,000 people is in danger of contracting HCC. Countries with limited medical and social care have a higher prevalence of HCC cases. In the USA, HCC is the most rapidly rising cause of death among all cancers with 42,030 new cases and 31,780 new deaths annually, and its management is still difficult^2–4^. Although liver transplantation provides patients diagnosed with HCC with the best outcomes, there is a paucity of donor organs. HCC prognosis is affected by its level of severity level at the time of diagnosis. Early diagnosis coupled with optimal medical management can save the native liver and alleviate the need for donor organs ^5,6^.

In addition to current physical examination and blood test, imaging techniques (e.g., magnetic resonance imaging (MRI), computed tomography (CT), etc.)^3,7^ are widely used to detect and grade HCC liver tumors. The American College of Radiology (ACR) supports the Liver Imaging Reporting and Data System (LI-RADS) to develop a standard for imaging interpretation and reporting in patients with cirrhosis or other risk factors for HCC^8^. In LI-RADS, liver tumors are classified as LR1 (definitively benign), LR2 (probably benign), LR3 (indeterminate), LR4 (probably HCC), LR5 (definitively HCC), or LRM (malignant but not definitely HCC). LI-RADS was formally launched in 2011 and, due to its importance to medical practice worldwide, it had four updates, most recently in 2018^9^. Although LI-RADS criteria provides high specificity^3,7^, it might produce low sensitivity due to subjectivity, especially if the liver tumor is classified as LR3, LR4, or LRM. Additionally, a recent meta-analysis suggests that HCC may be present at the following rates, depending upon LI-RADS classification: 0% in LR1, 13% in LR2, 38% in LR3, 74% in LR4, and 94% in LR5^9,10^, which might reduce certainty and objectivity of the final diagnostic decision. Biopsy serves as the last option to definitively diagnose liver cancer tumors. However, biopsies are invasive, expensive, and have the potential for adverse effects such as bleeding or infection^10,11^. Therefore, there is an urgent need for a non-invasive, objective, and accurate computer-aided diagnostic (CAD) system to detect and grade liver tumors.

Recent advances in machine learning (ML) enabled many groups to investigate its potential in early detection and grading of liver cancer tumors, as ML algorithms can deal with large amount of data and extract distinguishing effective markers improving the diagnosis accuracy^12–15^. Sato et al.^16^ conducted a study for the diagnosis of HCC. They worked with clinical biomarkers obtained from 1582 patients (HCC = 539 and non-HCC = 1043). Their ground truth was based on contast-enhanced CT scans. They investigated the power of multiple ML algorithms to produce the best diagnostic classifier. An accuracy of 87.34% for detecting malignant HCC tumors was obtained with gradient boosting. However, their technique did not incorporate any imaging features to capture differences between HCC and non-HCC tumors. In addition, the grading of these detected HCC tumors for proper management plan was not investigated. Yang et al.^17^ utilized contrast-enhanced MR (CE-MR) images to differentiate between malignant, intermediate, and benign liver tumors. Their study included 51 liver tumors (nine malignant, 35 intermediate, and seven benign). First, they manually delineated 2D ROIs on the liver tumors using image rendering software. Then, they input these ROIs to a multichannel fusion, three-dimensional convolutional neural network (CNN). Although they reported an accuracy of 91%±3% in detecting the malignant group alone, they achieved a lower average accuracy of 74%±1% in differentiating the three groups from each other.

Stocker et al.^18^ explored the power of texture analysis to differentiate benign from malignant HCC tumors. Their study included 108 patients (malignant HCC = 55 and benign = 53) with preoperative 2D CE-MRI. After placing 2D manual ROIs, they selected 13 textural markers. Then, they employed a binary logistic regression analysis on the extracted markers and analyzed them using statistical tests. Their model differentiated between malignant HCC and benign tumors with an accuracy of 84.5% using the arterial phase images. Although logistic regression demonstrated good diagnostic abilities in the differentiation between benign and malignant HCC, the authors did not explore their system’s performance on intermediate-grade HCC tumors. Yamashitaet et al.^19^ utilized multi-phase CT and MRI scans to classify liver tumors. They allocated 314 patients (CT = 163 and MRI = 151) with liver tumors and classified them according to the LI-RADS system as the ground truth into four categories (LR1–2 = 89, LR3 = 62, LR4 = 65, and LR5 = 98). For each subject, they selected one 2D image from each phase (4 images/subject). Using manual ROIs, these images were cropped and resized. Then, the resulting images were fed to a transfer learning CNN model and a custom CNN model. They obtained a better accuracy of 60.4% in differentiating between different LI-RADS categories (LR1-2, LR3, LR4, and LR5) using the transfer learning model. Subsequently, they validated this CNN on two external datasets (CT = 68 and MRI = 44) and achieved an accuracy of 47.7% and 41.2% for the external MRI and CT datasets, respectively. Their study was limited by only including a single image from each phase to represent the whole subject, which might lead to missing important morphological and anatomical features about the tumor.

Kim et al.^20^ focused on liver tumors of 41 patients undergoing CE-MRI scans and were categorized as (LR3 = 12, LR4 = 3, and LR5 = 26) to develop and evaluate a threshold-based CAD system for classification of the risk of HCC. After performing a semi-automated segmentation, three features were calculated: tumor size (maximum diameter), appearance of wash-out, and capsule appearance. Then, receiver operating characteristic (ROC) curves were constructed to determine the best threshold for each feature. Subsequently, they estimated the intraclass correlation coefficients and performed a statistical *t*-test to compare their computed markers to the reference radiologists’ markers. Their CAD system had a classification agreement of 76%:83% of the tumors. Their study was limited by excluding LR1 and LR2 patients. However, they did not investigate ML methods to enhance the classification performance. Moreover, the two reference radiologists only had an agreement of 78%. Wu et al.^21^ conducted a study using multiphase CE-MRI obtained from 89 patients with liver tumors to discriminate intermediate (LR3 = 35) from combined malignant (LR4 = 14 and LR5 = 40). They placed 2D manual ROIs on the center image per phase and used as input to a pre-trained AlexNet CNN model. Their model accomplished a 90% accuracy in the classification between the two groups. This study was limited by excluding the benign tumors. Additionally, they did not consider grading these tumors into LR4 and LR5.

**Figure 1:**
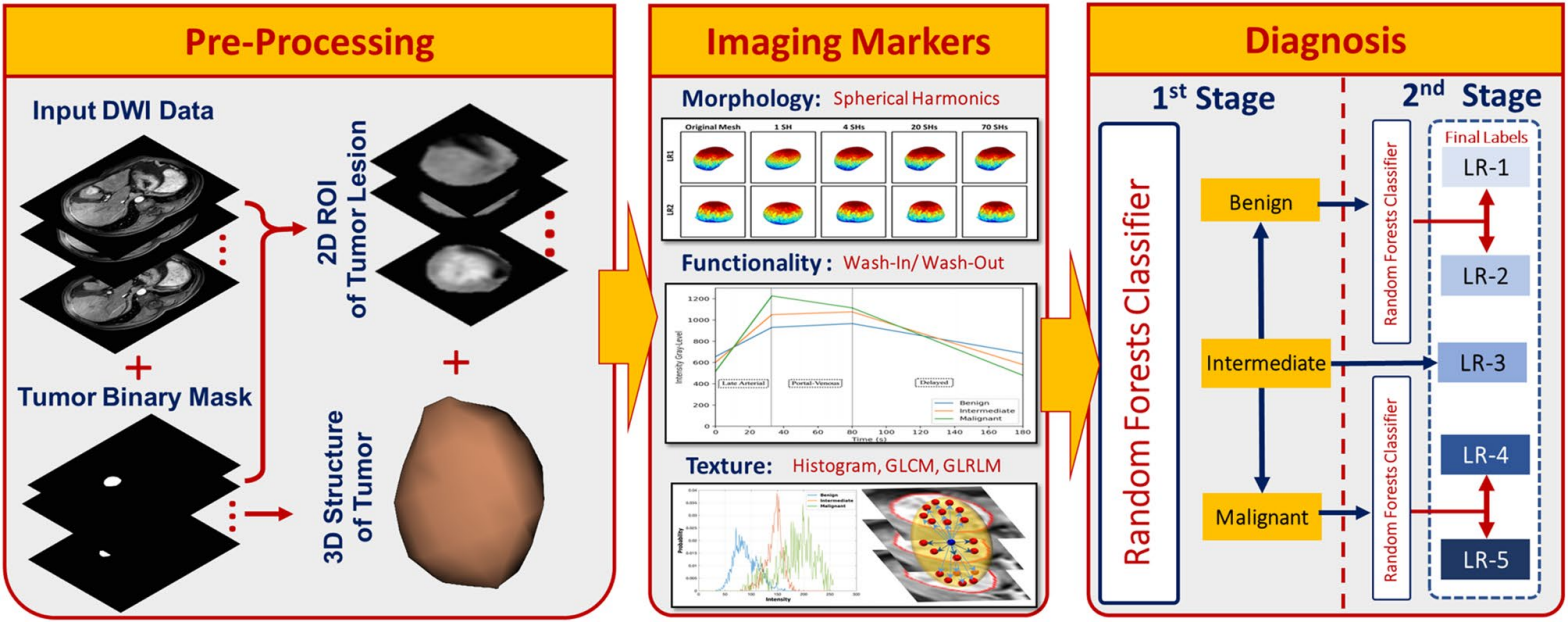
The proposed computer-aided diagnosis (CAD) system for detecting and grading liver cancer tumors.

However, most of the aforementioned studies only investigated the ability of individual textural markers or clinical records along with ML to differentiate between different liver tumors. A few studies investigated the grading of the liver tumor itself, which is critical for administering the proper treatment at an early stage, but were limited by their low diagnostic performance. In addition, none of these studies integrated morphological markers with first and second order textural markers and functional markers to provide an accurate diagnosis. To overcome these limitations, we have developed a novel two-stage computer-aided diagnostic (CAD) system with the ability to (*i*) differentiate between benign, intermediate, and malignant HCC liver tumors and (*ii*) discriminate between different grades of malignant and benign tumor sub-types. A schematic illustration of the proposed framework is shown in Fig. 1. To the best of our knowledge, the developed CAD system is the first of its kind to integrate novel morphological markers with rotation invariant textural markers and functional markers to differentiate malignant from intermediate, and benign tumors and determine the grade of the tumor to enable optimal medical management.

## Materials

*Study design and patients population* Liver tumor patients with a high risk of developing HCC without a history of loco-regional treatment plan were included in this study. Patients with cirrhosis, chronic hepatitis, and patients with prior HCC were included. For multiple liver tumors in the same patient, separate analyses were performed for each tumor. The methods were carried out in accordance with relevant guidelines and regulations. All experimental protocols were approved by the University of Louisville, USA and Mansoura University, Egypt. Contrast-enhanced MR images were obtained for 97 participants in the period between November 2018 and January 2021. All participants were fully informed about the aims of the study and provided their informed consents. However, two patients were excluded from the study due to withdrawal of consent. The remaining 95 patients with liver tumors *(M = 65 and F = 30)* ranged in age from 34 to 82 years old (average 56 y ± 10 y). Using a secondary work station (Phillips Advantage windows workstation with functional tool software), three expert radiologists, blinded from each other, with more than 10 years of hands-on experience in liver imaging analyzed all CE-MR images of all participants according to LI-RADS v2018^5^. The image analysis was performed for four major markers including: nonrim arterial phase hyper-enhancement (APHE); non-peripheral wash-out appearance; enhancing capsule appearance; and size of the liver tumor. For each subject, three decisions were provided and the final decision was taken based on an agreement of at least two of them. Among the participating patients, 38 liver tumors were diagnosed as benign tumors (LR1 = 19 and LR2 = 19), 38 were diagnosed as malignant tumors (LR4 = 19 and LR5 = 19) and 19 were diagnosed as intermediate (LR3) tumors. More details about the characteristics of the participating patients are documented in Supplementary 2, Table B.1.

*MR data acquisition protocol* CE-MR images were obtained for the aforementioned patient population (N = 95) using a 1.5T Philips Ingenia scanner with phased-array torso surface coil. Extracellular contrast agent (gadolinium chelates) with a dose of 0.1 mmol/kg was injected at rate of 2 ml/s using an automated MR injector followed by a 20 ml saline flush. The abdomen MR scanning includes four different phases: pre-contrast (at *t* = 0 s), late arterial (at *t* = 35 s), portal venous (at *t* = 50 s), and delayed-contrast phase (at *t* = 180 s). All patients were asked to hold their breath during image acquisition to minimize possible respiratory effects. MRI acquisition parameters are summarized in Table 1.

**Table 1:** Acquisition parameters of CE-MRI sequences.

CE-MRI Acquisition Parameters						
TR (ms)	TE (ms)	FOV (mm)	Slice size (pixels)	Slice thickness (mm)	Slice gap (mm)	Flip angle
7.3	3.1	500	256$\times$128	3	1	$40^\circ$

TR: repetition time; TE: echo time; FOV: field of view.

## Methods

The proposed CAD system to detect and grade liver cancer tumors is illustrated in Fig. 1. The CAD system performs the following steps: (*i*) extract morphological markers from the segmented liver tumors by using a new parametric spherical harmonic model, (*ii*) calculate textural markers that have been estimated by using a novel rotation invariant models, (*iii*) estimate the functional markers that have been calculated by estimating the wash-in/wash-out slopes to quantify the enhancement characteristics across different CE-MR phases, and (*iv*) model a two-stage random forest-based classification using the fusion of the identified markers to classify the liver tumor to benign, intermediate, or malignant and its corresponding grade (LR1, LR2, LR3, LR4, or LR5).

### Features/markers extraction

The features/markers extraction step is a core component of the machine learning pipeline. A marker in machine learning is an independently measurable property or attribute of an observation. Selecting good markers that clearly distinguish between object classes increases the predictive power of the machine learning model. So, this process aims to reduce the raw data into standardized, distinctive, and machine understandable markers that the learning algorithm can use to solve the main classification problem. In consultation with our medical collaborators, we had decided upon several categories of markers that are suited to the nature of our problem. Three different types of markers are extracted from the segmented liver tumors to provide a quantitative discrimination between different types and grades of liver tumors, namely: (*i*) morphological markers based on spherical harmonics (SH) that have the ability to describe the morphology complexity of the liver tumors, (*ii*) functional markers based on the calculation of the wash-in/wash-out slopes to quantify the enhancement characteristics across different phases, and (*iii*) textural markers, namely; the first-order histogram markers, novel rotation invariant second-order markers based on gray-level co-occurrence matrix (GLCM) and gray-level run-length matrix (GLRLM), to capture texture differences between different types and grades of liver tumors.

### Imaging markers

**Figure 2:**
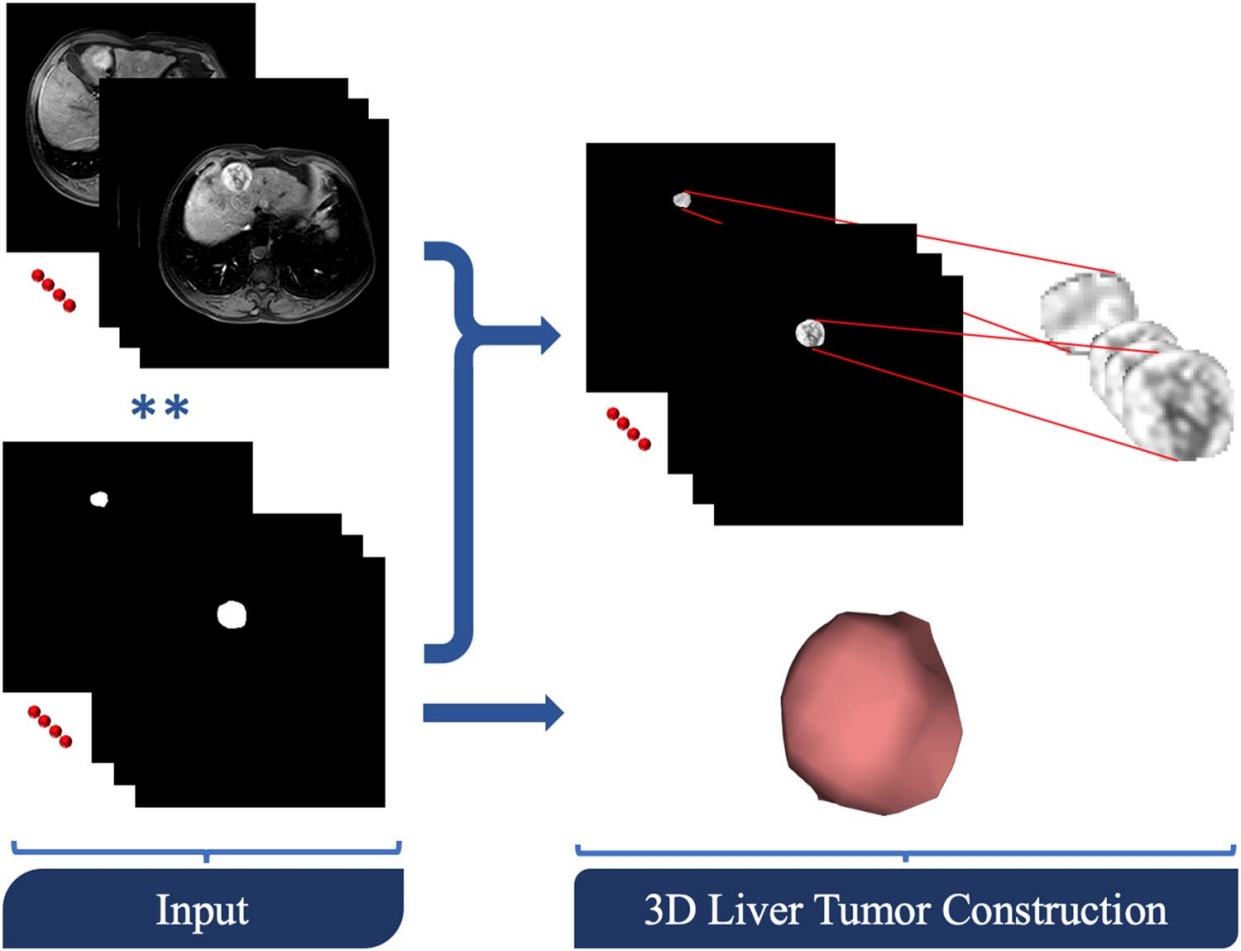
Liver tumors segmentation and 3D objects construction.

In order to enhance the performance of extracting/estimating morphological, textural, and functional imaging markers, all liver tumors were manually and accurately segmented using in-house software by two expert radiologists with more than 10-years of hands-on experience in medical image analysis, and consequently, 3D liver tumors objects were constructed (Fig. 2). To provide a precise discrimination between different types and grades of liver tumors, we characterized liver tumors objects by three different types of distinguishing image markers, namely; morphological markers, textural markers, and functional markers. These markers are described below in detail.

**Figure 3:**
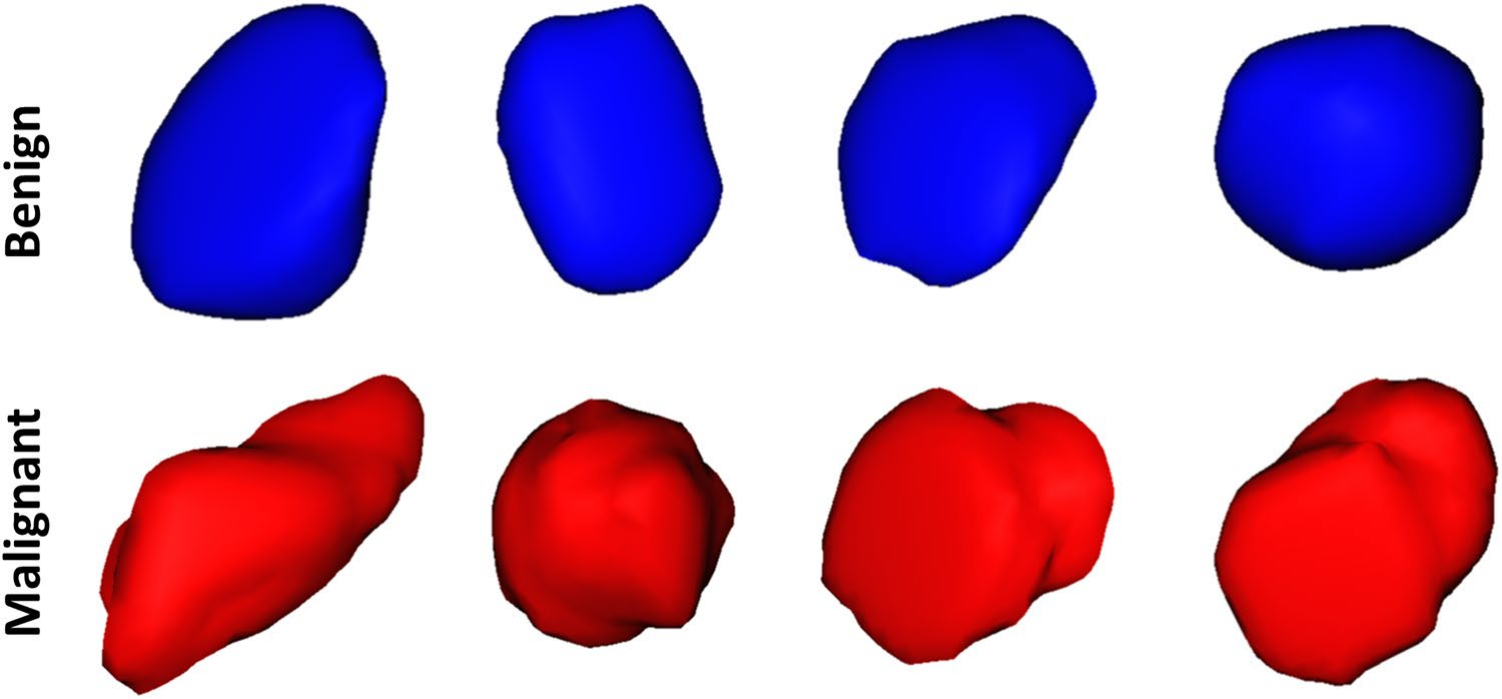
3D visualization of the surface complexity differences between benign tumors (blue) and malignant ones (red).

Morphological Markers: To improve the sensitivity and specificity of early liver cancer diagnosis, new parametric morphology markers that can describe the complexity of the detected liver tumor were identified. The motivation for using morphological markers relies on the hypothesis that malignant tumors have greater growth rates and more complex shapes than benign tumors. As demonstrated in Fig.3, the morphology and surface complexity of liver tumors vary based on the malignancy status and its corresponding grade. The utilization of the morphology description will enhance the automated diagnosis capabilities. However, accurate modeling is critical in achieving such enhancement. In the proposed framework, we used the state-of-the-art spectral analysis employing spherical harmonics (SH)^22^ to extract morphological markers for diagnosing liver tumors. Choosing a point inside the tumor as the origin of a spherical coordinate system, the tumor’s surface may be considered a function of polar and azimuthal angle, which can be expressed as a linear combination of basis functions $Y_{\tau \beta }$ defined on the unit sphere. The SH modeling builds a triangulated mesh approximating the tumor’s surface, then maps it to the unit sphere. The mapping approach, using an attraction-repulsion technique^23^, provides precise modeling, as it keeps unit distance between each re-mapped node and the origin, while preserving distances between neighboring nodes.

Let $\mathbf {C}_{\alpha ,i}$, with $\left\| \mathbf {C}_{\alpha ,i}\right\| = 1$, be the coordinates of node *i* on iteration $\alpha$ of the attraction-repulsion algorithm, where $i \in \{1, \ldots , I\}$. Let $\mathbf {d}_{\alpha ,ji}=\mathbf {C}_{\alpha ,j} - \mathbf {C}_{\alpha ,i}$ denote the displacement from node *i* to node *j*, so the Euclidean distance between nodes *i* and *j* is $d_{\alpha ,ji} = \left\| \mathbf {d}_{\alpha ,ji}\right\|$. Finally, let $J_i$ denote the index set of neighbors of node *i* in the triangulated mesh. Then the attraction step updates the position of each node to keep it centered with respect to its neighbors:

1 $$\begin{aligned} \mathbf {C}_{\alpha +1,i}^{\prime } = \mathbf {C}_{\alpha ,i}+C_{\mathrm {A},1}\sum \limits _{j\in J_i}\left( \mathbf {d}_{\alpha ,ji} d_{\alpha ,ji}^2 + C_{\mathrm {A},2} \frac{\mathbf {d}_{\alpha ,ji }}{d_{\alpha ,ji}}\right) , \end{aligned}$$

where attraction factors $C_{\mathrm {A},1}$ and $C_{\mathrm {A},2}$ are parameters of the algorithm. The repulsion step subsequently inflates the whole mesh to ensure that it does not become degenerate, as the attraction step by itself would allow nodes to become arbitrarily close to one another.

2 $$\begin{aligned} \mathbf {C}^{\prime \prime }_{\alpha +1,i}=\mathbf {C}^{\prime }_{\alpha +1,i} + \frac{C_{R}}{2I} \sum _{j=1;j\ne i}^{I} \frac{\mathbf {d}_{\alpha ,ji}}{\mathbf {d}_{\alpha ,ji}^{2}}, \end{aligned}$$

where, repulsion factor $C_{\mathrm {R}}$ is once again a parameter of the algorithm. Finally, the points are projected back onto the unit sphere, $\mathbf {C}_{\alpha +1,i} = \mathbf {C}_{\alpha +1,i}^{\prime \prime } / \Vert \mathbf {C}_{\alpha +1,i}^{\prime \prime }\Vert$.

At the terminal iteration $\alpha _f$ of the Attraction Repulsion algorithm, the surface of the liver nodule is in a one-one correspondence with the unit sphere. Each node $\mathbf {C}_i = (x_i, y_i, z_i)$ of the original mesh has been mapped to a corresponding point $\mathbf {C}_{\alpha _f,i} = (\sin \theta _i \cos \phi _i, \sin \theta _i \sin \phi _i, \cos \theta _i)$ with polar angle $\theta _i\in [0,\pi ]$ and azimuthal angle $\phi _i\in [0,2\pi )$. It then becomes possible to describe the nodule by an SH series. In this representation, lower order harmonics give the rough extent of the nodule, while higher order harmonics provide the finer details of the surface. The SHs are generated by the solving an isotropic heat equation for the nodule surface considered as a function on the unit sphere. The SH $Y_{\tau \beta }$ of degree $\tau$ and order $\beta$ is defined as:

3 $$\begin{aligned} Y_{\tau \beta }={\left\{ \begin{array}{ll} c_{\tau \beta }G_{\tau }^{\left| \beta \right| }\cos \theta \sin (\left| \beta \right| \varphi ) &{} -\tau \le \beta \le -1 \\ \frac{c_{\tau \beta }}{\sqrt{2}}G_{\tau }^{\left| \beta \right| }\cos \theta &{} \beta =0 \\ c_{\tau \beta }G_{\tau }^{\left| \beta \right| }\cos \theta \cos (\left| \beta \right| \varphi ) &{} 1\le \beta \le \tau \end{array}\right. } \end{aligned}$$

where $c_{\tau \beta }$ is the SHs factor and $G_{\tau }^{\left| \beta \right| }$ is the associated Legendre polynomial of degree $\tau$ and order $\beta$.

**Figure 4:**
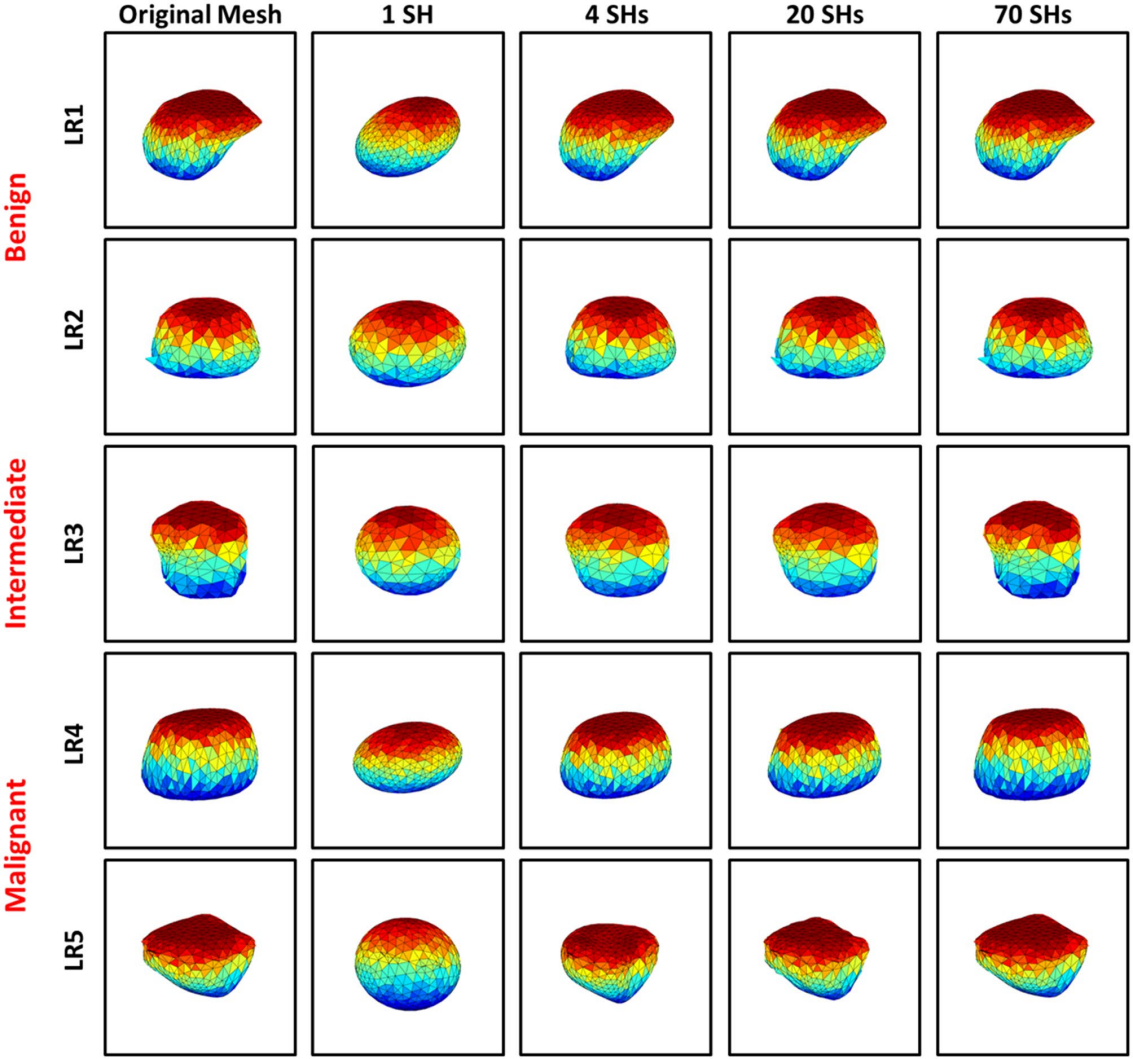
Morphology approximation of two benign (LR1-2), intermediate (LR3), and two malignant (LR4-5) tumors.

Finally, the liver tumor object is reconstructed/approximated from the SHs of Eq. 3. Benign tumors are represented using a lower order combination of SHs as their morphology are less complex, while malignant tumors are represented using higher-order combination of SHs as their morphology are more complex. Therefore, the total number of markers quantifying the morphological complexity of the detected tumors is the number of the SHs used to reconstruct the original tumor. In this study, the sufficient number (70) is used to correctly reconstruct any tumor, and after which there are no significant changes in the approximations. For each approximation, the reconstruction error between the original mesh and the approximated shape is calculated. Due to the unit sphere mapping, for each approximation, the original mesh for the tumor is inherently aligned with the mesh of the approximate shape, and the sum of the Euclidean distances between the corresponding nodes gives the total error between both the mesh models. By calculating this for the 70 approximations of each tumor, 70 numerical values (reconstruction errors) are obtained, which quantitatively describe the morphology of the tumor. Figure 4 shows the morphology approximation for five liver tumors (two benign, two malignant, and one intermediate). Summary of the Attraction-Repulsion algorithm is provided below.

- Initialization:

- Triangulate the surface of the nodule.
- Smooth the triangulated mesh with Laplacian filtering.
- Initialize the spherical parameterization with an arbitrary, topology-preserving map onto the unit sphere.
- Fix values of $C_{\mathrm {A},1}$, $C_{\mathrm {A},2}$, $C_{\mathrm {R}}$, and threshold *T*.
  - Attraction-repulsion:
- For $$\alpha = 0, 1, \ldots$$
  - For $$i = 1, \ldots , I$$
    - Calculate $\mathbf {C}_{\alpha +1,i}^{\prime }$ using Eq. 1
  - For $$i = 1, \ldots , I$$
    - Calculate $\mathbf {C}_{\alpha +1,i}^{\prime \prime }$ using Eq. 2
    - Let $$\mathbf {C}_{\alpha +1,i} = \mathbf {C}_{\alpha +1,i}^{\prime \prime } / \Vert \mathbf {C}_{\alpha +1,i}^{\prime \prime }\Vert$$
  - If $\max _{i} \Vert \mathbf {C}_{\alpha +1,i} - \mathbf {C}_{\alpha ,i}\Vert \le T$ Then let $\alpha _f = \alpha + 1$ and Stop.

*Textural markers* To improve the sensitivity and the specificity of early liver cancer diagnosis, a comprehensive textural analysis was performed. In particular, first and second order textural markers that can describe the inhomogeneity/homogeneity of the detected liver tumor were extracted from the four different phases/sequences, namely; pre-contrast, late arterial, portal venous, and delayed-contrast phase.

**Figure 5:**
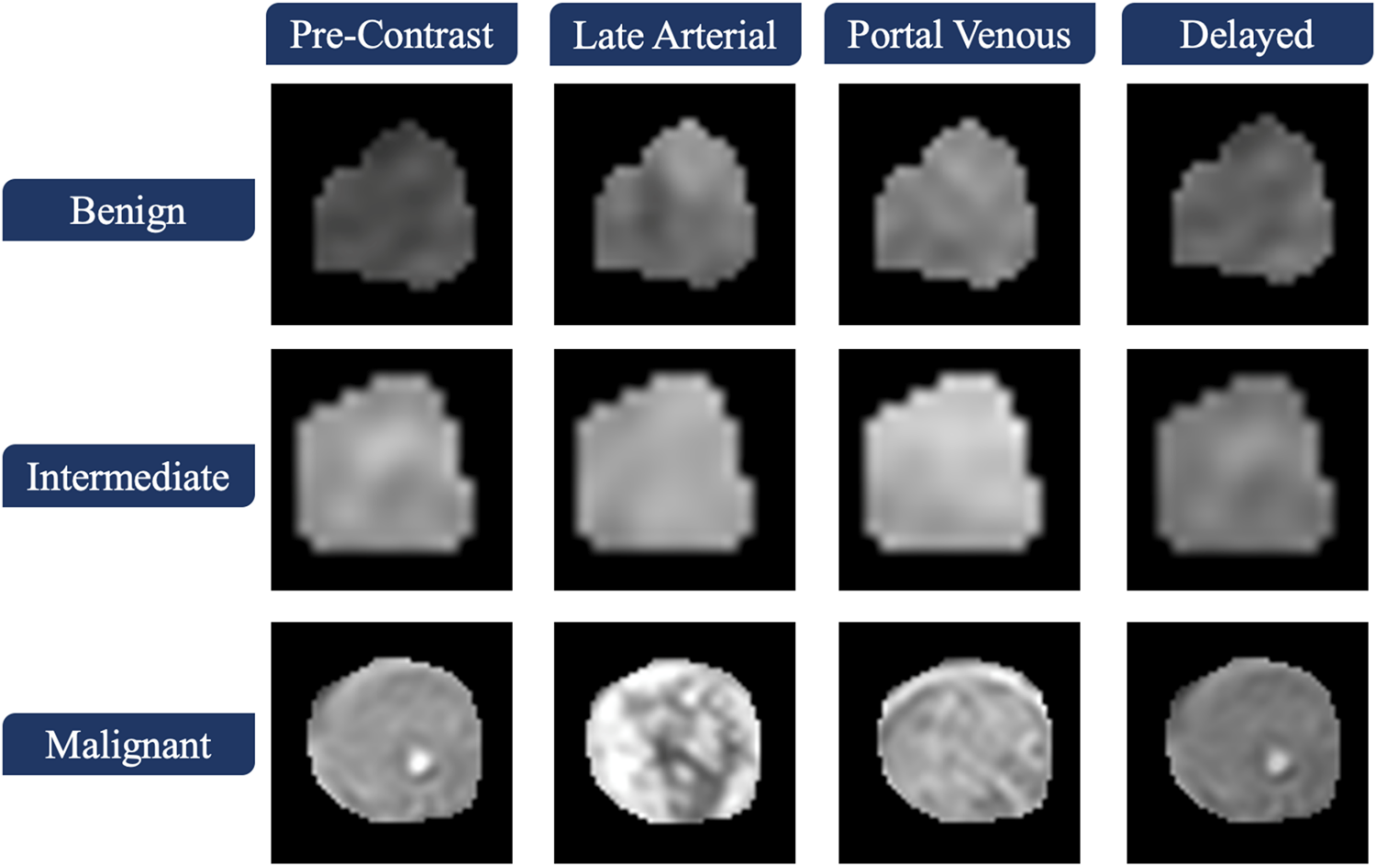
Texture differences visualization between three different tumors, (benign, intermediate, and malignant) at the four different phases.

The motivation for using textural markers relies on the hypothesis that malignant tumors appearance is inhomogeneous compared to benign tumors^24–30^. Figure 5 demonstrated the differences in inhomogeneity between benign and malignant tumors which supported our hypothesis.

**Figure 6:**
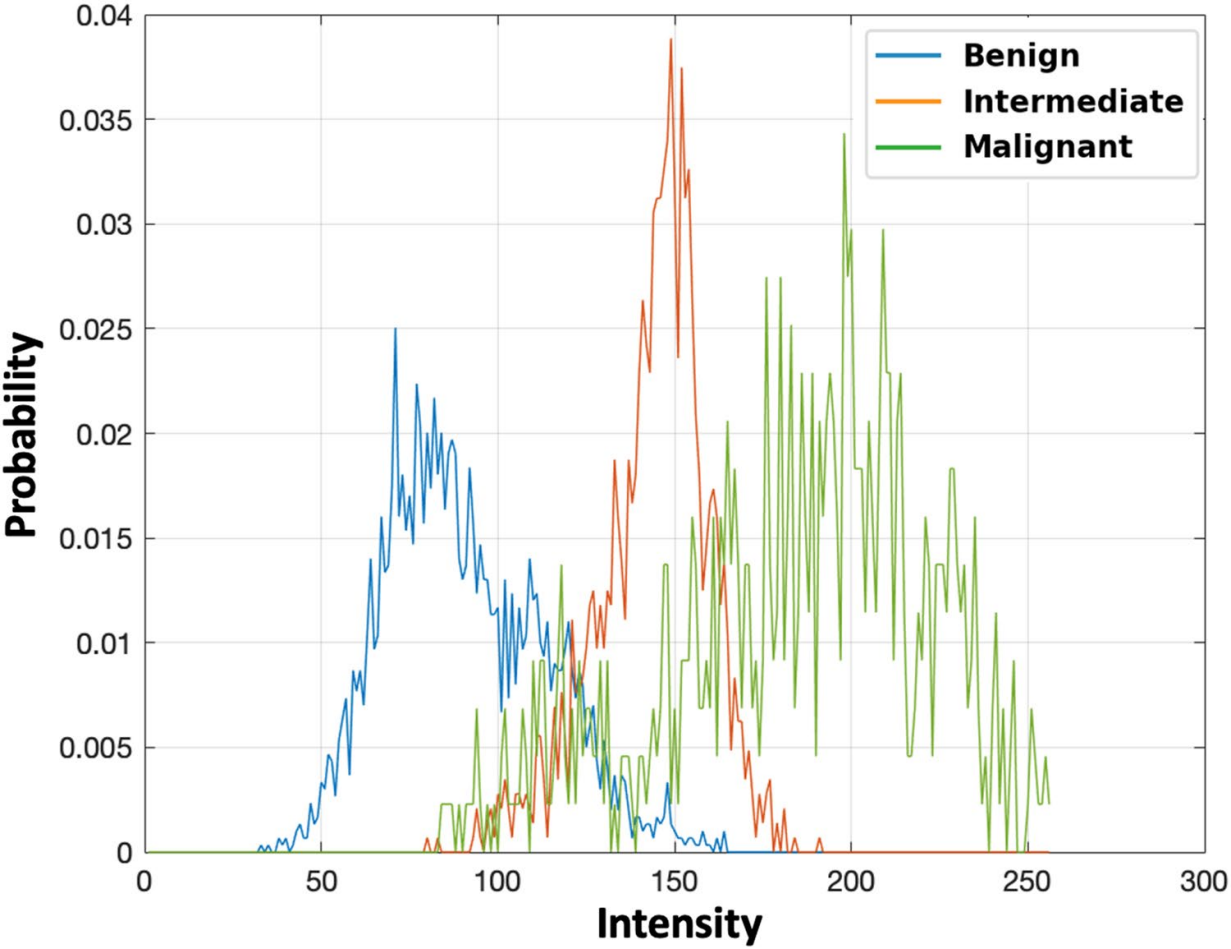
Probability density function visualization of the normalized gray-level intensity histogram for three different liver tumors: benign (blue), intermediate (orange), and malignant (green).

For the first order, a normalized empirical histogram (Fig. 6) was used to estimate all the first-order textural markers that are shown in Table 2^31^. The mathematical formulas of these markers are summarized in Supplementary 1, Table A1.

Since the first order texture might be sensitive to noise, two types of second order textural markers (gray-level co-occurrence matrix (GLCM) and gray-level run-length matrix (GLRLM)) were used to capture the inhomogeneity in liver tumors^32,33^.

**Figure 7:**
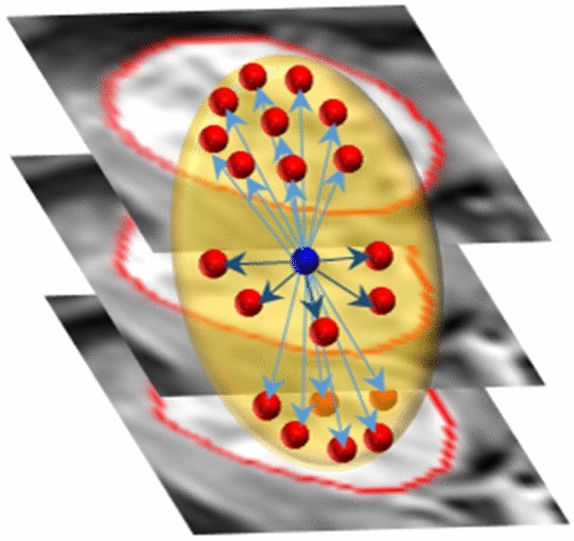
Visualization of rotation invariant neighborhood system of the center voxel (blue) to construct GLCM.

**GLCM:** is a matrix that considers the spatial relationships between voxels (the reference and the neighboring voxels) at a neighborhood block. Specifically, GLCM accounts for how frequently a pair of gray-level intensity values appears adjacently within the object. These frequencies are calculated for all gray-level possible pairs according to the gray-level range of the targeted object. The construction of the GLCM starts with specifying the range of gray-levels of the object and normalizing observed gray-level values to the desired range. Then all possible pairs are determined representing the matrix rows and columns (each element within the matrix is related to two gray-level values representing the row and the column of this element). Finally, the value of each element in the matrix is computed by examining how each voxel is different from its neighbors. The neighborhood block is defined by a distance $\le \sqrt{2}$ making the calculations rotation invariant as shown in Fig. 7. During analysis, gray-level values were normalized to the range of [0, 255], yielding a GLCM with size of 256$\times$256.

After constructing the GLCM, the matrix is normalized such that the sum of all elements is 1 in order to extract the discriminating textural markers^31,32^. Table 2 shows these markers. The reader is referred to Supplementary 1, Table A2 for the equations used to obtain these markers.

**GLRLM:** In addition to calculating the frequency of occurrence of voxel pairs represented by GLCM, GLRLM measure the voxels’ connectivity by looking at voxel runs. It examines how many times each gray-level value appeared consecutively in a run of voxels. This matrix has its number of rows equal to the gray-level range and number of columns as the largest possible run which is the largest dimension of the object (typically appears in the *XY*-Plane). Hence, each element in the matrix indicates the frequency of a specific gray-level value (the element’s row index) in a specific run length of consecutive voxels (the element’s column index). Each structure had a matrix with 256 rows (normalized gray-level range) while the number of columns is different amongst objects. Here, we looked for runs of consecutive horizontal voxels in the *XY*-Plane (in the same layer) and vertical run of voxels is examined in the *Z*-Plane (among different layers). Then, distinguishing measures of the GLRLM describing the texture of our structures were computed^31,33^. These markers are shown in Table 2. The reader is referred to Supplementary 1, Table A3 for the equations used to obtain these markers.

**Table 2:** First and second order textural markers.

Textural marker	Definition
First order	
Mean ($\mathrm {\mu }$)	Represents the gray-level values balance point of each object. It is calculated simply by getting the average gray-level value for each object.
Variance	Describes the gray-level distribution around our computed Mean.
Skewness	Expresses how the gray-level values are asymmetrically distributed around the Mean of the object.
Kurtosis	Measures to what extent the gray-level values are concentrated towards the tails of the distribution.
Entropy	Expresses the amount of randomness within each structure gray-level values.
CDFs	Return the cumulative distribution function of the histogram density values. This is calculated along the whole object and getting the cumulative sum of the gray-level values (Normalized to [0 to 1] at multiple positions (from 0 to 100% of the object with a 10% step).
Percentiles	Calculate the percentiles of gray-level values for the corresponding CDFs.
Second order	
Contrast	Measures the disparity in gray-level values between neighbors.
Dissimilarity	Finds to what extent voxels are different from their neighbors.
Homogeneity	Expresses the inverse difference moment among neighbors.
Angular second moment (ASM)	Determines the gray-levels local uniformity (orderliness).
Energy	The square root of the ASM.
Correlation	Determines the gray-level linear dependency between center voxel and its neighbors.
Gray-level non-uniformity (GLN)	Describes the dissimilarity of gray-level values within the object.
High gray-level run emphasis (HGLRE)	Measures the concentration of high gray-level values in the structure.
Long run emphasis (LRE)	Determines how long run lengths are distributed in the object indicating the coarseness of the texture.
Long run high gray-level emphasis (LRHGLE)	Measures how long runs of high gray-level values are distributed in the object.
Long run low gray-level emphasis (LRLGLE)	Measures how long runs of low gray-level values are distributed in the object.
Low gray-level run emphasis (LGLRE)	Measures the concentration of low gray-level values in the structure.
Run entropy (RE)	Indicates the amount of randomness in gray-level runs in the structure.
Run length non-uniformity (RLN)	Expresses the inhomogeneity among run lengths in the object.
Run percentage (RP)	Is calculated by the division of the overall count of runs by the total number of pixels.
Short run emphasis (SRE)	Measures the concentration of short run lengths in the object indicating how fine the texture is.
Short run high gray-level emphasis (SRHGLE)	Measures the concentration of high gray-level values short runs in the object.
Short run low gray-level emphasis (SRLGLE)	Measures the concentration of low gray-level values short runs in the object.

### Functional markers

**Figure 8:**
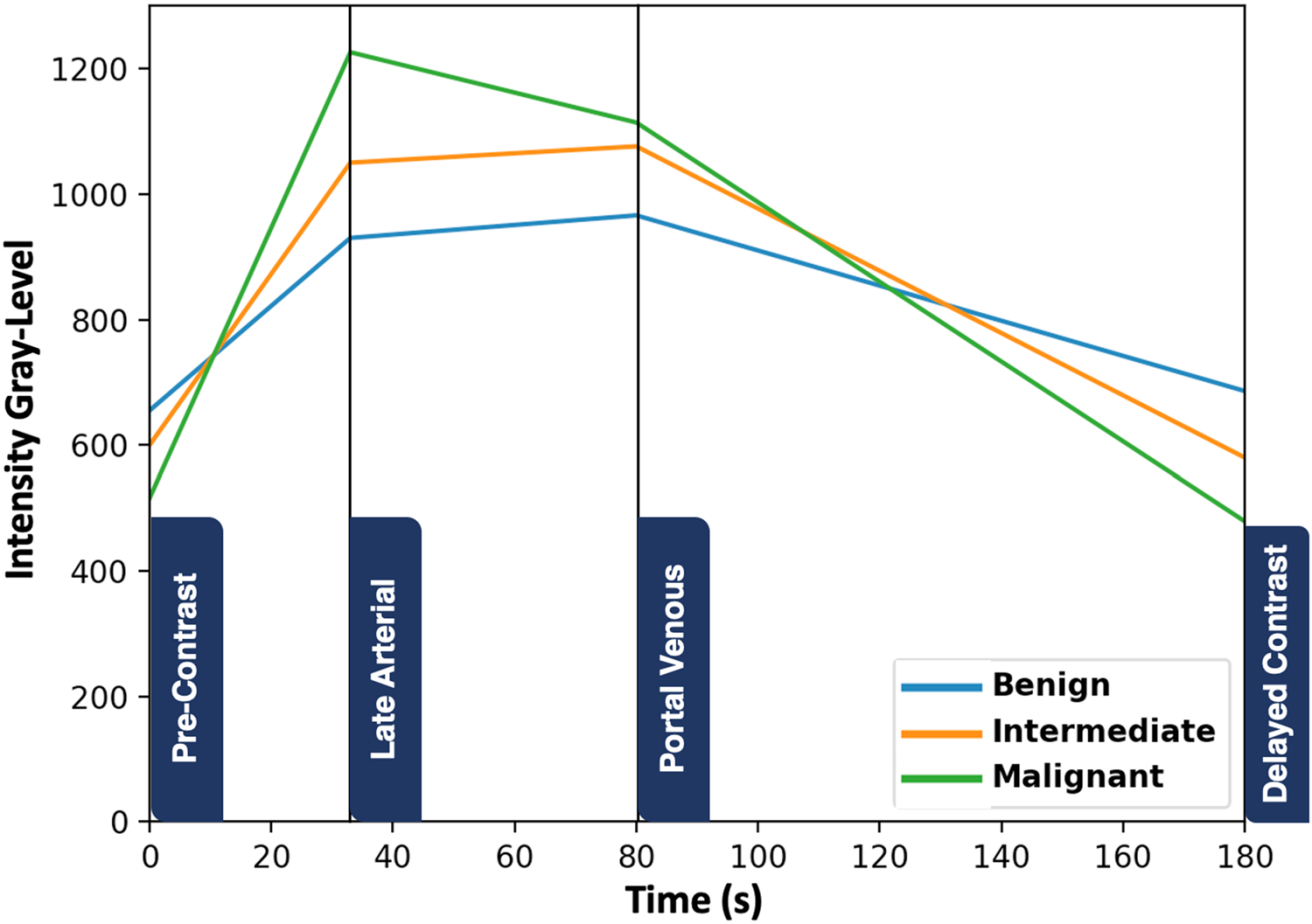
Typical wash-in/out slopes for three different tumors, (benign shown in blue, intermediate in orange, and malignant in green).

Liver tumor’s functionality can be quantified by hyperenhancement (wash-in) and hypointensity (wash-out). The wash-in can be estimated in the late arterial phase while the wash-out is estimated in the portal venous phase and/or delayed phase^34,35^. To compute the functional markers, we studied the gray-level intensity changes across the post contrast phases extracting three features. These features are mathematically expressed by the gray-level slope in each phase. These slopes are calculated by getting the gray-level intensity change rate over the time of each phase. Typically, positive slopes for wash-in and negative for wash-out. Malignant tumors have a higher and more rapid wash-in and wash-out slopes than those of intermediate or benign tumors. Figure 8 shows the wash-in and wash-out slopes, for a malignant, an intermediate, and a benign tumor during the three post-contrast phases.

### Features/markers selection

Features/markers selection is a method of selecting the most desirable and appropriate characteristics from a large collection of potential markers. This process results in *m* markers chosen out of a set of *n* possibilities, where $m < n$, and *m* is the smallest set of significant and important markers. Two approaches were applied here, namely, Wrapper approach^36,37^ and Gini impurity-based selection^38^.

*Wrapper approach* The selection process in wrapper methods is based on repeatedly running a particular machine learning algorithm on a given dataset. Comparing the results of the algorithm, provided various marker subsets on input, the wrapper method selects the combination of markers giving optimum performance. Note the specific performance criterion depends upon the problem being solved. The wrapper method follows a greedy search strategy through the space of possible markers. We performed two different wrapper approaches to find the optimal set of markers: (*i*) Forward Selection: Beginning with a null model, single-feature models are fitted one at a time, and the marker with the lowest *p*-value is chosen as optimal. Each of the remaining markers is combined with the one previously selected in a two-parameter model, and the additional marker with the lowest *p*-value is again chosen. Then each remaining marker is combined in turn with the previous two to find the third optimal marker, and so forth. Forward selection thus generates models with $1, 2, \ldots , m$ markers, terminating when none of the remaining candidate markers have a *p*-value less than a predetermined threshold. Algorithm  1 summarizes the forward selection approach. Here, we applied the forward selection with two significance thresholds (0.05 and 0.1). (*ii*) Bi-directional elimination (Step-wise Selection): It is similar to forward selection, but the difference is that it also tests the importance of already added markers before introducing a new one, and if it considers any of the already selected markers irrelevant, this marker is simply eliminated. The steps of this approach are shown in Algorithm  2. Here, we also applied the bi-directional elimination with two thresholds of significance (0.05 and 0.1). 
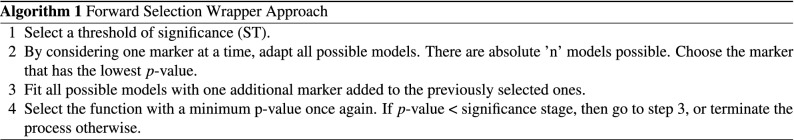

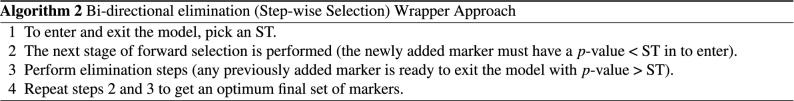


*Gini impurity-based selection* In a data science workflow, Random Forests are also used for features/markers selection. This resulted from the fact that the tree-based approaches used by random forests naturally rely on how well the purity of the node is enhanced. This suggests a drop in impurity over all trees, called Gini impurity. At the start of the trees, nodes with the largest decrease in impurity occur, while nodes with the least decrease in impurity occur at the end of the trees. Thus, we can build a subset of the most significant markers by pruning trees below a given node. Algorithm 3 shows the steps of applying this selection approach. To apply this algorithm, we performed the selection process in two different scenarios (combined and separate markers selection). For the combined selection, we applied the Gini impurity-based approach on the whole set of markers to find the optimal set of markers to use. While for the separate method, we performed the selection on the morphological, textural, and functional markers separately to find the optimal markers at each group. Then, we combined these limited markers sets to build the final, optimal marker set. 
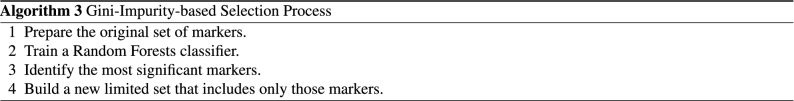

**Table 3:** Illustration of different categories and their associated number of extracted markers for each subject.

Morphological markers	
Spherical Harmonics	70 markers
Textural markers	
First order (Histogram markers)	104 markers (26/phase)
Second order (GLCM)	24 markers (6/phase)
Second order (GLRLM)	48 markers (12/phase)
Functional markers	
Wash-In/Out	3 markers
Integrated markers	
Combined	249 markers

### Liver tumor markers integration and diagnosis

After extracting the discriminating markers (morphological, functional, and textural) for all liver tumors at the four different phases, a two stage classification process is used to obtain the final diagnosis of these tumors. The first stage targets differentiating between benign (LR1-2), intermediate (LR3), and malignant (LR4-5) tumors. The second stage further classifies the benign tumors into either LR1 or LR2, and classifies the malignant tumors into either LR4 or LR5. Several well-known ML classifiers were used (e.g., random forests (RFs), fine k-nearest neighbor (kNN$_{Fine}$), support vector machine (SVM) with cubic kernel (SVM$_{Cub}$), SVM with quadratic kernel (SVM$_{Quad}$), naive Bayes (NB), and linear discriminant analysis (LDA)). First, classification performance was assessed using individual markers, namely, SHs morphological markers, the first order textural markers, the second order GLCM textural markers, the second order GLRLM textural markers, and wash-in/wash-out slopes functional markers. The categorized numbers and description of these discriminating markers is detailed in Table 3. Subsequently, all the markers were integrated by using concatenation methods obtaining combined markers. The aforementioned ML classifiers were used for the final diagnosis. A grid search algorithm along with the diagnostic accuracy as an optimization metric were employed to find the optimal set of different ML classifiers’ hyper-parameters. The optimal sets of hyper-parameters for each classifier are as follows: RFs (class weight=’balanced’, criterion=’gini’, max depth=30, min samples leaf=5, min samples split=2, n estimators=100), kNN$_{Fine}$ (leaf size=30, metric=’minkowski’ with power of 2, n neighbors=5, weights=’uniform’), SVM (regularization parameter = 1, break ties=False, cache size=200, decision function shape=’ovr’, degree=3, gamma=0.001, max iter=-1, tol=0.001), NB (alpha=0.5, binarize=0.0, class prior=None, fit prior=True), and LDA (n components=1, priors=None, shrinkage=0.52, solver=’lsqr’, store covariance=False, tol=0.0001).

Given a liver tumor CE-MR series, one can obtain the final diagnosis (LR1, LR2, LR3, LR4, or LR5) of that tumor by applying the developed CAD system steps outlined in Algorithm 4 below. 
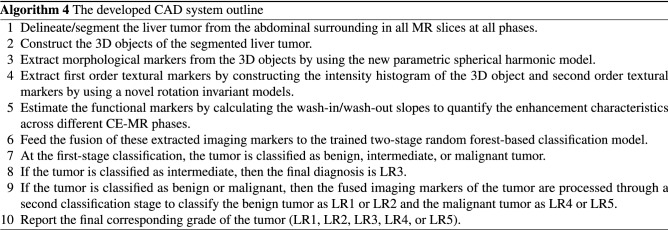


## Experimental results

The diagnostic accuracy of the proposed CAD system was evaluated using a leave-one-subject-out (LOSO), randomly stratified 10-fold, and randomly stratified 5-fold cross-validation approaches. LOSO relies on training the classification model with all observations except one subject set aside for testing purposes. The classification model is then reinitialized before the next iteration, and the observation previously left out is included in the training data, leaving the following subject out for testing purposes. This process is repeated for 95 times (i.e., the total number of subjects in our dataset), and at each iteration, the training and the testing samples are of size 94 and 1, respectively. For the stratified *k*-fold cross-validation, a fraction ${1\over k}\times 100\%$ of the data are randomly selected and left for the testing purposes, while the remaining ${{1 - k}\over k}\times 100\%$ part of data are used as the training data. The classification model is then reinitialized in the next iteration, and the subjects left in the previous iteration are included in the training, leaving the next ${1\over k}\times 100\%$ part of subjects aside for testing purposes. This process is repeated for *k* iterations. To assure the robustness of the developed model, we performed the randomly stratified *k*-fold cross validation approach with two values of *k*, i.e. 10 and 5.

It is important to keep in mind that in the implementation of *k*-fold cross-validation, stratification was guaranteed to help reduce both bias and variance. The technique of stratification not only enables randomization, but also ensures that the training/testing sets would have the same proportion of each class as in the entire data set. In our case, stratification means that 40% of the training/testing sets will be derived from benign subjects (N = 38), 20% from intermediate (N = 19), and 40% from malignant cases (N = 38).

Two classification stages were performed to obtain the final diagnosis. In order to quantitatively express the classification performance, each classification process was repeated 10 times and the obtained results were reported in terms of mean±standard deviation. The first classification stage aimed to differentiate between benign (LR1–2), intermediate (LR3), and malignant tumors (LR4–5). The performance of the developed CAD system was first assessed using the obtained individual markers, namely; morphological markers, textural markers, and functional markers along with several ML classifiers. To highlight the advantage of integrating these individual markers, we compared the diagnostic performance of the combined model with these individual models using the following metrics: sensitivity, specificity, and F$_{1}$ score^39,40^,

4 $$\begin{aligned} Sensitivity= & {} \frac{TP}{TP+FN} \end{aligned}$$

5 $$\begin{aligned} Specificity= & {} \frac{TN}{TN+FP} \end{aligned}$$

6 $$\begin{aligned} F_{1}~score= & {} \frac{2TP}{2TP+FP+FN} \end{aligned}$$

where TP is the number of correctly classified malignant subjects; TN is the number of correctly classified benign subjects; FP is the number of benign and intermediate subjects misclassified as malignant; and FN is the number of malignant and intermediate subjects misclassified as benign. The combined model achieved sensitivity of 91.8%±0.9%, specificity of 91.2%±1.9%, and F$_{1}$ score of 0.91±0.01 using the RFs classifier outperforming the performance of all individual models as shown in Table 4. This enhanced diagnostic performance due to the integration process enables the algorithm to account for different aspects of quantifying markers (morphological, textural, and functional).

**Table 4:** Comparison of the first stage classification performance using the individual markers namely, SHs morphological markers, First order textural markers, second order GLCM textural markers, second order GLRLM textural markers, and wash-in/out slopes functional markers of the developed CAD system: Benign (LR1-2) vs. Intermediate (LR3) vs. Malignant (LR4-5) using RFs classifier. Note that: Sens and Spec denote Sensitivity and Specificity, respectively.

Markers	Sens%	Spec%	${F} _{1}{} {score}$
**Morphological markers**			
Spherical Harmonics	73.39±3.13	84.43±1.98	0.78±0.02
**Textural markers**			
First Order (Histogram)	79.72±3.81	85.91±2.39	0.82±0.03
Second Order (GLCM)	86.45±2.17	87.93±1.76	0.86±0.01
Second Order (GLRLM)	81.94±2.30	81.96±2.38	0.81±0.01
**Functional markers**			
Wash-In/Out	81.11±2.71	84.37±1.85	0.82±0.02
**Integrated markers**			
Combined	91.81±0.88	91.17±1.90	0.91±0.01

**Table 5:** Comparison of the first stage classification performance Using the integrated markers of the developed CAD system: Benign (LR1-2) vs. Intermediate (LR3) vs. Malignant (LR4-5) using different machine learning classifiers and three validation approaches for each classifier (i.e. LOSO, 10-Fold, and 5-Fold). Let Sens, Spec, RFs, KNN, SVM, NB, and LDA denote sensitivity, specificity, random forests, k-nearest neighbor, support vector machine, naive Bayes, and linear discriminant analysis, respectively.

Classifier	Approach	Sens%	Spec%	F$_{1}$score
RFs	**LOSO**	**91.81**±**0.88**	**91.17**±**1.90**	**0.91**±**0.01**
	**10-Fold**	**88.88**±**0.94**	**90.38**±**2.52**	**0.89**±**0.02**
	**5-Fold**	**87.01**±**1.82**	**89.30**±**2.55**	**0.88**±**0.02**
kNN$_\mathbf{Fine }$	LOSO	86.24±0.00	91.89±0.00	0.89±0.00
	10-Fold	84.69±1.88	91.91±1.43	0.88±0.01
	5-Fold	83.41±2.29	90.56±2.79	0.87±0.02
${SVM }_{Cub, Quad}$	LOSO	84.62±0.00	86.84±0.00	0.86±0.00
	10-Fold	83.90±2.71	85.23±1.54	0.84±0.02
	5-Fold	83.99±2.89	85.23±1.54	0.84±0.02
NB	LOSO	82.86±0.00	86.84±0.00	0.84±0.00
	10-Fold	79.72±3.66	88.69±3.45	0.83±0.02
	5-Fold	79.36±4.07	88.78±3.43	0.83±0.03
LDA	LOSO	86.49±0.00	87.50±0.00	0.86±0.00
	10-Fold	82.77±4.62	85.96±1.84	0.84±0.03
	5-Fold	80.85±5.31	81.88±3.49	0.80±0.03

To find the optimal classifier for the developed CAD system, we compared the obtained diagnostic results of the combined model using several ML classifiers (i.e., RFs, KNN$_{Fine}$, SVM$_{Cub, Quad}$, NB, and LDA) along with different validation approaches (LOSO, 10-fold, and 5-fold). With sensitivity, specificity, and F$_{1}$ score of 91.8%±0.9%, 91.2%±1.9%, and 0.91±0.01, respectively, for the LOSO, 88.9%±0.9%, 90.4%±2.5%, and 0.89±0.02, respectively, for the 10-fold, and 87.0%±1.8%, 89.3%±2.6%, and 0.88±0.02, respectively, for the 5-fold, the RFs proves itself as the best among the used different ML classifiers. Table 5 summarizes the comparison results between the performances of different ML classifiers and approaches. The classification performance obtained by RFs^41,42^ can be justified by that they are well-known robust machine learning classification techniques that have been widely used in solving medical classification problems^43^. RFs is an example of an ensemble learner which is built on bagging a collection of decision trees and random subspace method. This bagging mechanism helps to find all possible correlations between the decision trees in an ordinary bootstrap sample. When some markers are found to be strong predictors to target output, these markers will be selected in many decision trees and become correlated. Once the training process is performed, the final results are normally obtained by majority vote or model averaging mechanism^41,42^. RFs classifier was selected for use in the proposed CAD system as it outperformed all other classifiers that were tested.

**Figure 9:**
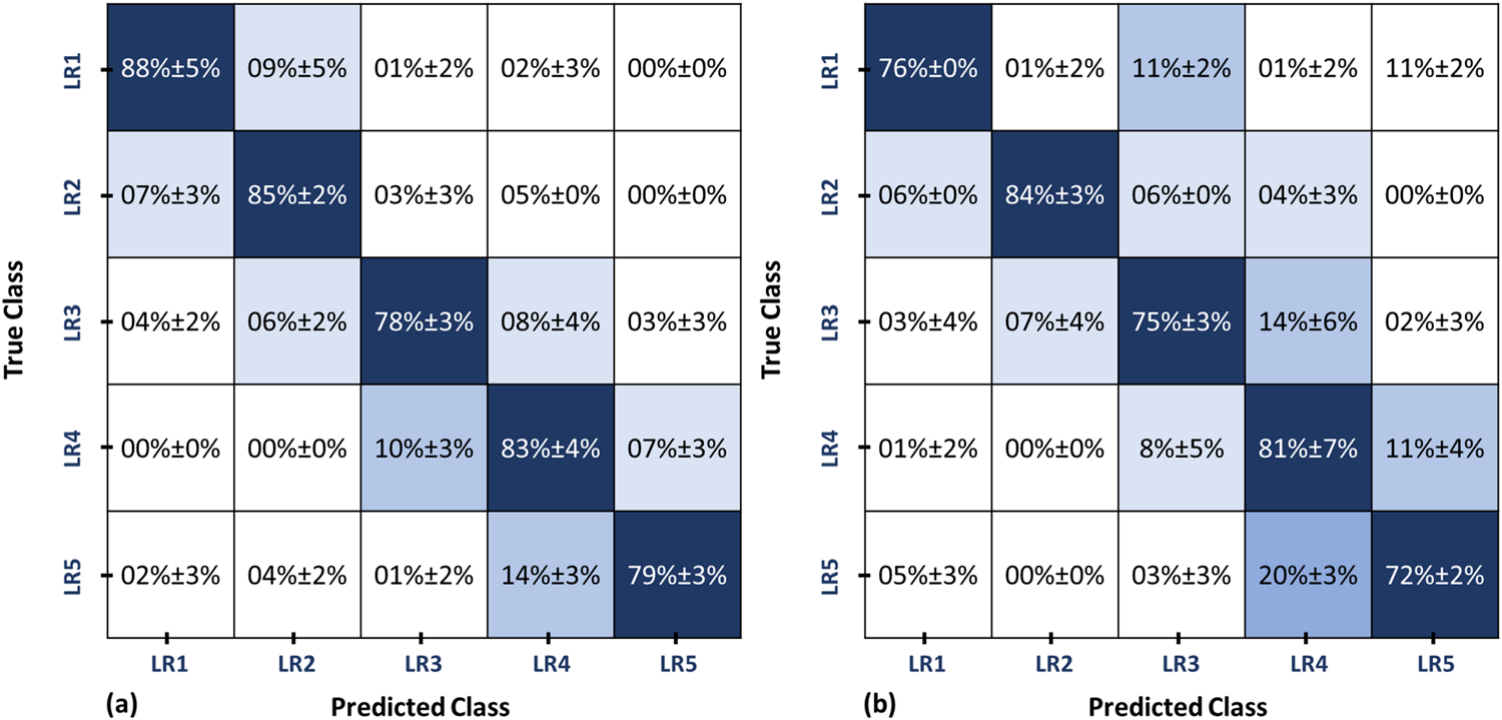
The overall confusion matrix obtained for the developed CAD system using LOSO approach utilizing the integrated markers for grading the tumors into (LR1, LR2, LR3, LR4, and LR5) using (**a**) a two-stage RFs classifier (proposed classification approach) compared to (**b**) a one-stage RFs classifier.

For the second classification stage, grading for each class was performed: benign class (LR1 vs. LR2) and malignant class (LR4 vs. LR5). All markers were combined together and fed to an RFs classifier to obtain the final diagnosis using LOSO, 10-fold, and 5-fold cross-validation approaches. As shown in Table 6 (using LOSO approach), an overall accuracy of 89.47±2.35% was obtained for grading the benign tumors, while 88.95±1.58% overall accuracy was obtained for grading malignant tumors. Finally, the results from both stages were combined to obtain the final diagnosis result, and grading of the tumors into LR1, LR2, LR3, LR4, and LR5. It is worth mentioning that the developed CAD system using a two-stage RFs classification model (see Fig. 1) provided more enhanced diagnostic performance than applying a single stage RFs classification as evidenced by the final confusion matrices shown in Fig. 9.

**Table 6:** Diagnostic Performance of the developed CAD system in the second stage classification: LR1 vs. LR2 and LR4 vs. LR5 using RFs classifier utilizing the combined markers.

Approach	Accuracy%		
Benign			
	LR1	LR2	Overall
LOSO	88.95±4.37	90.00±1.58	89.47±2.35
10-Fold	86.32±4.82	88.42±3.16	87.37±3.29
5-Fold	84.74±6.84	85.79±4.74	85.26±5.02
Malignant			
	LR4	LR5	Overall
LOSO	92.63±2.58	85.26±2.11	88.95±1.58
10-Fold	90.00±4.37	80.00±2.11	85.00±2.06
5-Fold	87.37±6.74	74.74±2.11	81.05±3.07

**Figure 10:**
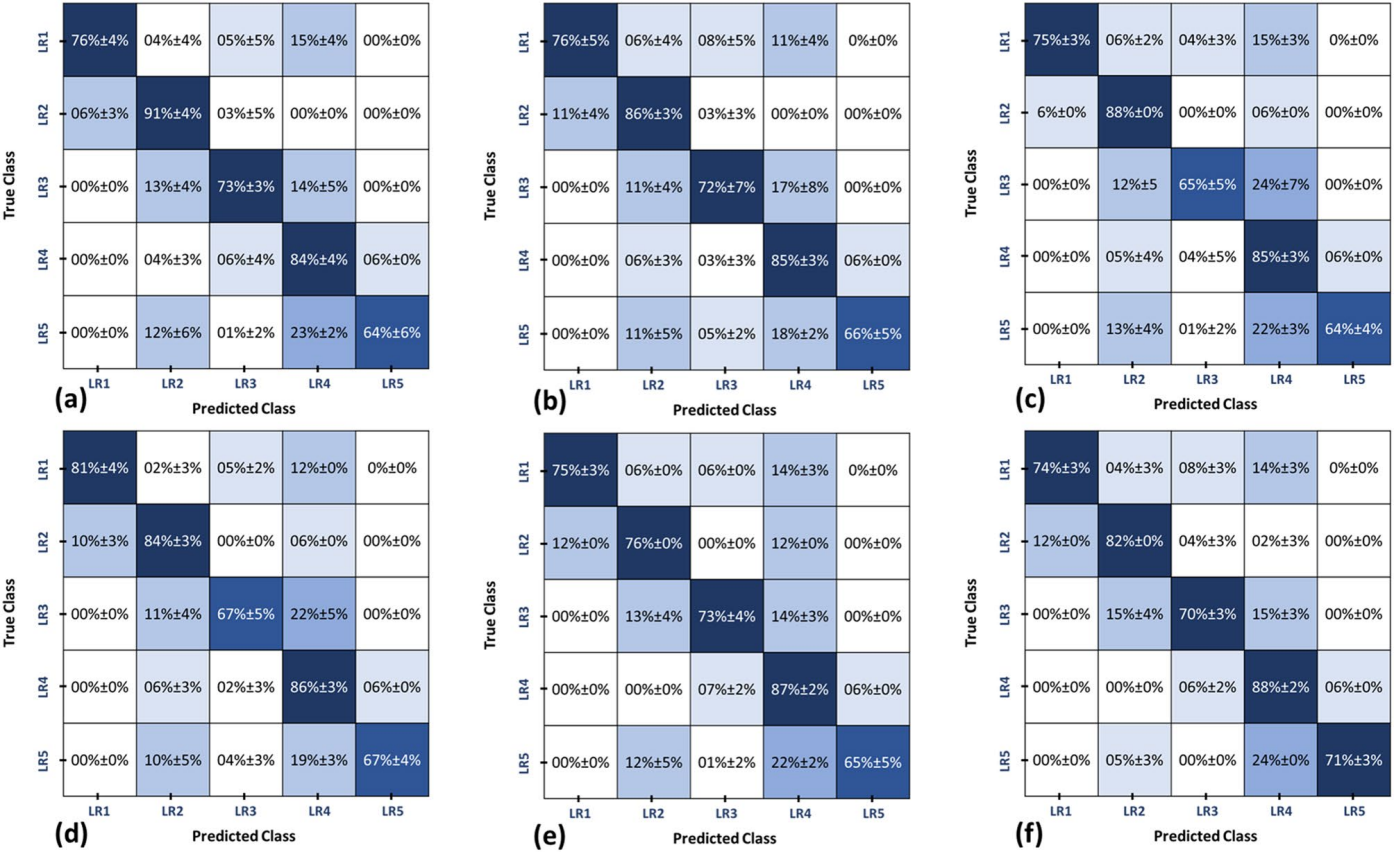
Confusion matrices comparing the final diagnostic performance obtained by using the proposed two-stage RFs classification model along with a LOSO approach for grading the tumors into (LR1, LR2, LR3, LR4, and LR5) after applying six different features/markers reduction techniques as follows: (**a**) wrapper approach based on bi-directional elimination (step-wise selection) using a significance threshold (ST) of 0.1, (**b**) wrapper approach based on bi-directional elimination (step-wise selection) using an ST of 0.05, (**c**) wrapper approach based on forward selection using an ST of 0.1, (**d**) wrapper approach based on forward selection using an ST of 0.05, (**e**) Gini impurity-based approach using combined selection, and (**f**) Gini impurity-based approach using separate selection.

To highlight the advantages of utilizing the integrated markers over the reduced markers, we compared the final diagnostic performance obtained by the developed CAD system with that obtained after applying six different scenarios of features/markers reduction, namely, (*i*) forward selection with ST = 0.05 (m = 19 markers), (*ii*) forward selection with ST = 0.10 (m = 196 markers), (*iii*) bi-directional elimination with ST = 0.05 (m = 13 markers), (*iv*) bi-directional elimination with ST = 0.10 (m = 16 markers), (*v*) Gini impurity-based selection on the combined markers (m = 134 markers), and (*vi*) Gini impurity-based selection on separate markers groups(morphological and textural markers) (m = 109 markers). In each scenario, we applied the proposed two-stage RF classification model on the output reduced markers to obtain the final diagnosis as (LR1, LR2, LR3, LR4, or LR5). The comparison results in terms of each individual LI-RAD accuracy and the overall accuracy are summarized in Table 7. For a favorable comparison, the complete confusion matrix of the developed CAD system is shown in Fig. 9(a) and the confusion matrices of the aforementioned scenarios are shown in Fig. 10.

**Table 7:** Comparison of the two-stage diagnostic performance using the developed CAD system (combined markers) with the performance of six different features/markers selection scenarios. Let m and ST denote the number of the used markers and significance threshold, respectively.

Markers selection approach		m	Accuracy%					
			LR1	LR2	LR3	LR4	LR5	Overall
Proposed CAD system (combined)		249	**88±5**	85±2	**78±3**	83±4	**79±3**	**83±2**
Wrapper Approach	forward (ST=0.05)	19	81±4	84±3	67±5	86±3	67±4	77±2
	forward (ST=0.10)	196	75±3	88±0	65±5	85±3	64±4	75±2
	bi-directional (ST=0.05)	13	76±5	86±3	72±7	85±3	66±5	77±2
	bi-directional (ST=0.10)	16	76±4	**91±4**	73±3	84±4	64±6	78±2
Gini-Impurity-based	combined selection	134	75±3	76±0	73±4	87±2	65±5	75±2
	separate selection	109	74±3	82±0	70±3	**88±2**	71±3	77±1

**Table 8:** The final diagnostic performance for grading the tumors into (LR1, LR2, LR3, LR4, and LR5) by using (a) the proposed CAD system, (b) approach by Stocker et al.^18^, and (c) approach by Wu et al.^21^.

Model	Accuracy%					
	LR1	LR2	LR3	LR4	LR5	Overall
Proposed CAD System	88±5	**85±2**	78±3	**83±4**	79±3	**83±2**
Stocker^18^	71±0	82±0	71±0	53±0	70±0	69±0
Wu^21^	**91±2**	58±9	**81±4**	60±8	**88±2**	76±4

**Figure 11:**
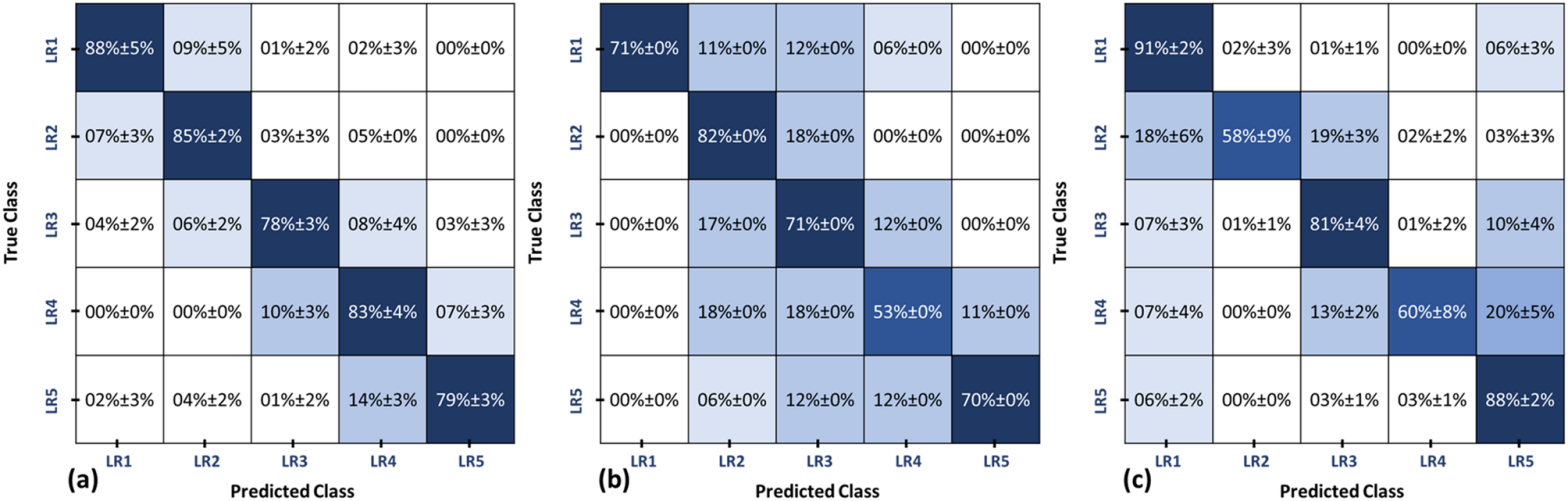
Confusion matrices comparing the final diagnostic performance for grading the tumors into (LR1, LR2, LR3, LR4, and LR5) by using (**a**) the proposed CAD sytem, (b) approach by Stocker et al.^18^, and (**c**) approach by Wu et al.^21^.

To appreciate the diagnostic performance obtained by the developed CAD system, we applied two different approaches from the literature^18,21^ on our dataset (N = 95) and the intended classification problem of liver tumor grading (LR1. vs. LR2. vs. LR3. vs. LR. vs. LR5) for a fair comparison. Then, we compared the final diagnostic results obtained by the developed CAD system with those obtained by the two different approaches. As documented in Table 8 and shown in Fig. 11, the diagnostic performance of developed CAD system outperformed all the aforementioned approaches for liver tumor grading.

## Discussion and conclusions

HCC has a high mortality at later stages. Effective identification of a comprehensive screening system at early stages is important and must be tailored to a broader algorithm for the management. Professional research groups have advocated recommendations to aid physicians and radiologists to handle HCC. LI-RADS aims to standardize the HCC-related lexicon and to create an image algorithm to boost the homogeneity of data collection and image reporting. The clinical gold standard for HCC diagnosis is image analysis performed by blinded independent expert radiologists for arterial phase hyperenhancement, wash-out appearance, enhancing capsule appearance, and size^3–9,44^.

On the other hand, radiogenomics and novel imaging developments are designed to understand HCC’s heterogeneity through imaging, and to facilitate individualized care for each tumor unique signature. Advanced algorithms and trends approved their ability to enable greater precision in diagnosis and grading, along with potential guidance on personalized health care^12–15,18–21,45–47^.

In this study, the extracted tumor lesions from the CE-MR images at different phases were combined in 3D objects. These 3D objects representing the subjects at different phases (4 phases per subject) consist of multiple voxels lying in the lesions and parenchyma of the surrounding liver. Each voxel displays a gray-scale value based on its signal strength which is influenced by the various histopathological factors. Therefore, in lesions, 3D arrays of gray-scale values may show complex geometric patterns that are distinctive to tumor forms, although they may be visually unrecognizable. For this reason, we performed texture analysis in our study. Texture analysis effectively describes how values of voxels depends on the gray-level of each voxel in a specific area. This texture information had proved itself to have great impact on the classification techniques performance in multiple studies^24–30^. In this study, we worked on first and second order texture analysis and extracted textural markers using different methods and algorithms. First order texture analysis explains how voxel intensities are distributed among tumor lesions at each phase. Thus, these descriptors depend basically on the independent value of each voxel. The computed first order markers are mean, variance, standard deviation, skewness, kurtosis, entropy, cumulative distribution functions, and gray-level percentiles^31^. Second order texture analysis algorithms vary from those first order algorithms in that they are essentially based on the neighborhood relationship between voxels. Such algorithms are spatially variant which implies that voxel arrangements relative to each other (neighbors) directly influence the analytical techniques of these algorithms. We have previously worked with both GLCM and GLRLM ^32,33^.

These GLCM and GLRLM second order texture analysis has shown an ability to differentiate between benign, malignant, and intermediate liver tumors due to its sensitivity to spatial interrelationships. The developed neoangiogenesis, high neovascularity and aggressive growth patterns within malignant tumors can cause complex internal architectures. This leads to a significant variation in micro-environment and heterogeneity between liver lesions with different malignancy status. Thus, more subtle variations in tumor heterogeneity can be identified by examining the voxel attenuation and its spatial interrelationships. Malignant tumor lesions show increased texture heterogeneity compared to intermediate and benign lesions. GLCM can determine if the voxels are uniformly distributed (Benign) or segregated in groups (malignant) and the GLRLM shows how these voxels are connected together across the whole lesion; long runs (homogeneous) or short runs (heterogeneous). All of these discrepancies could be observed, interpreted, and quantified using these extracted second order textural markers.

Furthermore, functional markers demonstrated a potential in identifying the malignancy status of a given liver tumor. Thus, we studied the gray-level intensity changes across the post contrast phases extracting three markers (late arterial wash-in, portal venous wash-out, and delayed wash-out). These markers are mathematically expressed by the gray-level slope in each phase. These slopes catch the variations in the enhancement markers that exist. In this analysis, the findings obtained through the measurement curves of functionality are fair and illustrate the efficacy of these markers in differentiating between different liver tumors’ grades.

A liver tumor’s grade of malignancy determines the morphology of the tumor. Malignant tumors usually show a more complex morphology than that of benign ones. Thus, morphological markers were used to identify potential variations between benign, intermediate, and malignant HCC tumors.

Liver tumors’ grades were identified by characterizing 3D objects structured from CE-MR images using morphological, textural, and functional markers. All markers were analyzed using machine learning models in the classification process. Although some of these markers showed substantial variations between different grades of liver tumors, there is still a large overlap. Such variation prevents the use of single markers class to better identify liver tumors, even though the most suitable CE-MR sequence has been used. Using a combination of markers provided a better approach to discriminating against malignant tumors from intermediate and benign ones. With significant diagnostic performance, the proposed system first distinguished between benign, intermediate, and malignant HCC tumors using the integration of all markers. Then using the same classification and validation processes, the LR1 benign tumors were classified from LR2, and LR4 malignant tumors were differentiated from LR5. Such findings reflect the accuracy of our methodology and the potential clinical utility of these approaches when used with CE-MR imaging in computer-aided diagnosis of liver tumors. These findings are documented in Tables 4, 5, and Fig. 9.

In conclusion, the developed CAD system demonstrated high diagnostic performance (sensitivity = 91.81%±0.88%, specificity = 91.17%±1.90%, and F$_{1}$ score = 0.91±0.01) by integrating morphological markers with textural markers and functional markers outperforming the diagnostic performance of each individual marker alone. In addition, the developed CAD system achieved overall accuracies of 88%±5%, 85%±2%, 78%±3%, 83%±4%, and 79%±3% in grading liver tumors into LR1, LR2, LR3, LR4, and LR5, respectively. These results demonstrates the feasibility of the integration process between different discriminating markers that account for different aspects of the liver tumor characteristics, namely; morphology, texture, and functionality. In the future, a larger subject cohort dataset will be used to further enhance the performance of the CAD system in distinguishing and grading multiple liver tumors. Additionally, other possible liver tumors with LRM will be added to our dataset to enhance the diagnostic abilities of the CAD system.

## Supplementary information

Supplementary file

## Data Availability

The datasets generated during and/or analyzed during the current study are available from the corresponding author on a reasonable request.
