# Supplementary material for: A novel computer-aided diagnostic system for accurate detection and grading of liver tumors
Source: Sci Rep. 2021 Jun 23;11:13148. doi: 10.1038/s41598-021-91634-0 (PMC8222341; doi:10.1038/s41598-021-91634-0)
Supplement: Supplementary file 1 — Supplementary file [file 41598_2021_91634_MOESM1_ESM.pdf]

Appendices

A

In this appendix, we are going to detail the mathematical formulas used to extract the textural markers and therefore, the following basic notations will be used:

Notation

- N: The largest possible run length.
- N_p: Gray-level intensity levels.
- N_g: Normalized intensity levels.
- n_r: The overall count of runs.
- n_p: The overall count of pixels.
- (i,j): Row and column index, respectively.
- P(i): Gray-level intensity values.
- g(i): Normalized gray-level intensity values.
- ε: Arbitrarily small, positive number
- GLCM: The gray-level Co-occurrence Matrix.
- CM: The normalized GLCM.
- CM(i,j): Element at position (i,j) in the normalized GLCM.
- RL: The gray-level Run Length Matrix.
- RL(i,j): Element at position (i,j) in the GLRLM.
- μ_{x}: The marginal rows mean.
- μ_{y}: The marginal columns mean.
- σ_{x}: The marginal rows standard deviation.
- σ_{y}: The marginal columns standard deviation.

**Attribute**	**Formula**
Skewness	$$\frac{\frac{1}{N_p}\sum_{i=1}^{N_p}(P(i)-\mu)^3}{\left(\sqrt{\frac{1}{N_p}\sum_{i=1}^{N_p}(P(i)-\mu)^2}\right)^3} \quad (1)$$
Kurtosis	$$\frac{\frac{1}{N_p}\sum_{i=1}^{N_p}(P(i)-\mu)^4}{\left(\frac{1}{N_p}\sum_{i=1}^{N_p}(P(i)-\mu)^2\right)^2} \quad (2)$$
Entropy	$$-\sum_{i=1}^{N_g} g(i)\log_2\left(g(i)+\varepsilon\right) \quad (3)$$

Table A.1. First order Histogram textural markers formulas

**Attribute**	**Formula**
Contrast	$$\sum_{i=0}^{N_g}\sum_{j=0}^{N_g}(i-j)^2 CM(i,j) \quad (4)$$
Dissimilarity	$$\sum_{i=0}^{N_g}\sum_{j=0}^{N_g}\lvert i-j\rvert CM(i,j) \quad (5)$$
Homogeneity	$$\sum_{i=0}^{N_g}\sum_{j=0}^{N_g}\frac{CM(i,j)}{1+(i-j)^2} \quad (6)$$
ASM	$$\sum_{i=0}^{N_g}\sum_{j=0}^{N_g}\left(CM(i,j)\right)^2 \quad (7)$$
Energy	$$\sqrt{ASM} \quad (8)$$
Correlation	$$\frac{\sum_{i=0}^{N_g}\sum_{j=0}^{N_g}CM(i,j)ij-\mu_x\mu_y}{\sigma_x(i)\sigma_y(j)} \quad (9)$$

Table A.2. Second order GLCM textural markers and their associated formulas.

Attribute	Formula
Gray-Level Non-Uniformity (GLN)	$$\frac{\sum_{i=0}^{N_g}\left(\sum_{j=0}^{N-1} RL(i,j)\right)^2}{n_r} \quad (10)$$
High Gray-Level Run Emphasis (HGLRE)	$$\frac{\sum_{i=0}^{N_g}\sum_{j=1}^{N-1} RL(i,j)i^2}{n_r} \quad (11)$$
Long Run Emphasis (LRE)	$$\frac{\sum_{i=0}^{N_g}\sum_{j=0}^{N-1} RL(i,j)j^2}{n_r} \quad (12)$$
Long Run High Gray-Level Emphasis (LRHGLE)	$$\frac{\sum_{i=0}^{N_g}\sum_{j=0}^{N-1} RL(i,j)i^2 j^2}{n_r} \quad (13)$$
Long Run Low Gray-Level Emphasis (LRLGLE)	$$\frac{\sum_{i=0}^{N_g}\sum_{j=0}^{N-1} \frac{RL(i,j)j^2}{i^2}}{n_r} \quad (14)$$
low gray-level run emphasis (LGLRE)	$$\frac{\sum_{i=0}^{N_g}\sum_{j=0}^{N-1} \frac{RL(i,j)}{i^2}}{n_r} \quad (15)$$
Run Entropy (RE)	$$-\sum_{i=0}^{N_g}\sum_{j=0}^{N-1} RL(i,j)\log_2 RL(i,j)+\varepsilon) \quad (16)$$
Run Length Non-Uniformity (RLN)	$$\frac{\sum_{j=0}^{N-1}\left(\sum_{i=0}^{N_g} RL(i,j)\right)^2}{n_r} \quad (17)$$
Run Percentage (RP)	$$\frac{n_r}{n_p} \quad (18)$$
Short Run Emphasis (SRE)	$$\frac{\sum_{i=0}^{N_g}\sum_{j=0}^{N-1} \frac{RL(i,j)}{j^2}}{n_r} \quad (19)$$
Short Run High gray-level Emphasis (SRHGLE)	$$\frac{\sum_{i=0}^{N_g}\sum_{j=0}^{N-1} \frac{RL(i,j)i^2}{j^2}}{n_r} \quad (20)$$
Short Run Low gray-level Emphasis (SRLGLE)	$$\frac{\sum_{i=0}^{N_g}\sum_{j=0}^{N-1} \frac{RL(i,j)}{i^2 j^2}}{n_r} \quad (21)$$

Table A.3. Second order GLRLM textural markers and their associated formulas.

B

In this appendix, we are showing information about cases included in this study, namely, gender, age, weight, Alpha-fetoprotein (AFP), risk factor, and diagnosis (ground truth):

Patient ID	Gender	Age	Weight	Tumor Marker (AFP)	Risk Factor	Diagnosis
01	F	52	65	30	Cirrhosis	LR-1
02	M	51	85	45	Cirrhosis	LR-1
03	F	48	55	14	Cirrhosis	LR-1
04	F	64	80	10	HBV	LR-1
05	M	53	95	75	HBV	LR-1
06	M	60	85	80	Cirrhosis	LR-1
07	M	45	85	20	HBV	LR-1
08	F	51	80	15	Cirrhosis	LR-1
09	M	51	85	25	HBV	LR-1
10	F	40	65	30	Cirrhosis	LR-1
11	M	53	85	40	Cirrhosis	LR-1
12	F	60	75	20	Cirrhosis	LR-1
13	M	36	75	11	HBV	LR-1
14	F	47	65	11	HBV	LR-1
15	M	51	80	15	Cirrhosis	LR-1
16	M	68	95	70	HBV	LR-1
17	M	42	70	90	Cirrhosis	LR-1
18	F	36	70	35	Cirrhosis	LR-1
19	M	66	80	60	Cirrhosis	LR-1
20	F	57	70	50	Cirrhosis	LR-2
21	M	82	67	15	HBV	LR-2
22	M	58	77	250	HBV	LR-2
23	M	49	85	40	HBV	LR-2
24	M	73	80	181	Cirrhosis	LR-2
25	F	65	80	90	Cirrhosis	LR-2
26	F	43	70	40	Cirrhosis	LR-2
27	M	60	80	25	Cirrhosis	LR-2
28	M	48	95	145	Cirrhosis	LR-2
29	M	75	70	104	Cirrhosis	LR-2
30	F	50	60	45	Cirrhosis	LR-2
31	M	52	80	310	Cirrhosis	LR-2
32	M	40	70	30	Cirrhosis	LR-2
33	M	67	95	40	Cirrhosis	LR-2
34	M	60	90	163	Cirrhosis	LR-2
35	M	58	80	150	Cirrhosis	LR-2
36	M	49	105	185	Cirrhosis	LR-2
37	F	45	74	195	Cirrhosis	LR-2
38	M	39	86	60	HBV	LR-2
39	M	62	72	160	HCV	LR-3
40	M	60	71	115	Cirrhosis	LR-3
41	M	63	75	315	HBV	LR-3
42	M	55	75	110	Cirrhosis	LR-3
43	M	53	88	120	Cirrhosis	LR-3
44	M	60	85	150	Cirrhosis	LR-3
45	M	63	90	260	Cirrhosis	LR-3
46	M	51	90	213	Cirrhosis	LR-3

Patient ID	Gender	Age	Weight	Tumor Marker (AFP)	Risk Factor	Diagnosis
47	F	64	80	110	HBV	LR-3
48	M	51	85	322	Cirrhosis	LR-3
49	M	59	80	95	Cirrhosis	LR-3
50	M	56	95	19	Cirrhosis	LR-3
51	F	46	80	55	Cirrhosis	LR-3
52	M	52	75	335	Cirrhosis	LR-3
53	F	45	80	60	Cirrhosis	LR-3
54	M	71	95	100	Cirrhosis	LR-3
55	M	51	75	90	Cirrhosis	LR-3
56	M	71	95	100	Cirrhosis	LR-3
57	F	79	56	220	Cirrhosis	LR-3
58	F	60	70	260	Cirrhosis	LR-4
59	M	57	65	520	Cirrhosis	LR-4
60	F	62	100	330	Cirrhosis	LR-4
61	M	47	70	210	Cirrhosis	LR-4
62	M	51	80	305	Cirrhosis	LR-4
63	M	73	80	180	Cirrhosis	LR-4
64	M	51	85	320	Cirrhosis	LR-4
65	M	51	80	370	Cirrhosis	LR-4
66	M	52	75	336	Cirrhosis	LR-4
67	M	56	80	300	Cirrhosis	LR-4
68	F	53	65	215	Cirrhosis	LR-4
69	F	69	55	300	Cirrhosis	LR-4
70	M	69	90	307	Cirrhosis	LR-4
71	F	53	75	130	HBV	LR-4
72	F	54	61	80	HBV	LR-4
73	M	53	60	95	Cirrhosis	LR-4
74	F	55	60	75	HBV	LR-4
75	F	64	75	120	Cirrhosis	LR-4
76	M	63	70	150	Cirrhosis	LR-4
77	M	49	77	251	HBV	LR-5
78	M	51	85	321	Cirrhosis	LR-5
79	M	63	70	400	Cirrhosis	LR-5
80	M	57	65	520	Cirrhosis	LR-5
81	M	61	70	420	Cirrhosis	LR-5
82	M	52	70	420	Cirrhosis	LR-5
83	M	51	80	371	Cirrhosis	LR-5
84	M	60	85	330	Cirrhosis	LR-5
85	M	52	75	337	Cirrhosis	LR-5
86	M	63	70	383	HBV	LR-5
87	F	34	60	308	Cirrhosis	LR-5
88	F	63	70	350	HBV	LR-5
89	F	36	55	410	HBV	LR-5
90	M	67	90	50	Cirrhosis	LR-5
91	M	60	95	320	HBV	LR-5
92	F	36	55	410	HBV	LR-5
93	M	80	70	310	Cirrhosis	LR-5
94	F	62	65	295	HBV	LR-5
95	M	64	112	10	HBV	LR-5

Table B.1. CE-MRI Data and Diagnosis.
